# A long-term outcome (up to 29 years) of bilateral iliac wings “bayonet osteotomies” for closure of bladder exstrophy

**DOI:** 10.1186/s13018-023-03810-9

**Published:** 2023-05-02

**Authors:** Jidapa Wongcharoenwatana, Nath Adulkasem, Thanase Ariyawatkul, Perajit Eamsobhana, Chatupon Chotigavanichaya, Areesak Chotivichit

**Affiliations:** Department of Orthopaedic Surgery, Faculty of Medicine Siriraj Hospital, Mahidol University, Bangkok, Thailand

**Keywords:** Bladder exstrophy, Pelvic osteotomy, Ectopia vesicae, Symphyseal closure, Symphyseal diastasis

## Abstract

**Background:**

Several types of pelvic osteotomy techniques have been reported and employed by orthopedic surgeons to enhance the approximation of symphyseal diastasis in bladder exstrophy patients. However, there is limited evidence on a long-term follow-up to confirm which osteotomy techniques provide the most suitable and effective outcomes for correcting pelvic deformities. This study aimed to describe the surgical technique of bilateral iliac bayonet osteotomies for correcting pelvic bone without using fixation in bladder exstrophy and to report on the long-term clinical and radiographic outcomes following the bayonet osteotomies.

**Methods:**

We retrospectively reviewed patients with bladder exstrophy who underwent bilateral iliac bayonet osteotomies with the closure of bladder exstrophy between 1993 and 2022. Clinical outcomes and radiographic pubic symphyseal diastasis measurements were evaluated. From a total of 28 operated cases, eleven were able to attend a special follow-up clinic or were interviewed by telephone by one of the authors with completed charts and recorded data.

**Results:**

A total of 11 patients (9 female and 2 male) with an average age at operation of 9.14 ± 11.57 months. The average followed-up time was 14.67 ± 9.24 years (0.75–29), with the average modified Harris Hip score being 90.45 ± 1.21. All patients demonstrated decreased pubic symphyseal diastasis distance (2.05 ± 1.13 cm) compared to preoperative (4.58 ± 1.37 cm) without any evidence of nonunion. At the latest follow-up, the average foot progression angle was externally rotated 6.25° ± 4.79° with full hips ROM, and no patients reported abnormal gait, hip pain, limping, or leg length discrepancy.

**Conclusions:**

Bilateral iliac wings bayonet osteotomies technique demonstrated a safe and successful pubic symphyseal diastasis closure with an improvement both clinically and radiographically. Moreover, it showed good long-term results and excellent patient’s reported outcome scores. Therefore, it would be another effective option for pelvic osteotomy in treating bladder exstrophy patients.

## Introduction

Bladder and cloacal exstrophy is a rare complex congenital malformation involving the genitourinary tract, the musculoskeletal tissue, and the gastrointestinal tract. It occurs in 1 in 10,000–50,000 live births [1–4]. Classic bladder exstrophy combines epispadias with a midline defect of the lower part of the abdominal wall and protruded bladder. The severity of malformations varies from epispadias, bladder exstrophy, and cloacal exstrophy. In order to yield better outcomes, these conditions must be treated appropriately with cooperation between orthopedists and pediatric urologists.

Characteristics of a bony pelvis in bladder exstrophy patients are an anterior deficiency with wide pubic diastasis and externally rotated innominate bones of the pelvis. Two-and three-dimensional (3D) CT has been used to characterize the exstrophic pelvis morphology, demonstrating an increased 30° of external pelvic rotation, combined between 12° of additional posterior segment external rotation and 18° of additional anterior segment external rotation, compared with normal. The deformity also showed 30% of shortening and increased 31% of interradiate distance [1, 5, 6]. These deformities resulted in pubic diastasis up to 4.2 cm at birth and can increase to 14.2 cm in adults [1, 7]. Consequently, patients can display externally rotated of foot progression angles around 20°–30° greater than normal accompanied by a wide-based gait associated with hip dysplasia [8, 9].

Accordingly, understanding those pelvic anatomical abnormalities is extremely important in order to plan proper surgical correction and a better prognosis. The main objective of pelvic reconstruction is to reduce pubic symphyseal diastasis and be able to close the bladder and abdominal wall while diminishing the soft tissue tension. Sponseller et al. [10] stated that closure of the bladder alone without pelvic osteotomy showed a higher incidence of premature hip arthrosis. Ideally, osteotomy for correcting pelvic anatomy must produce an internal rotation of the anterior and posterior pelvic segments with a lengthening of the anterior pelvic segment that can amend the inward tilt of both iliac wings and decrease interradiate distance [11]. Sponseller [12] developed a technique of bilateral anterior osteotomy of the innominate bone. However, this procedure was technically demanding and related to morbidity. Other previous studies on solely posterior pelvic osteotomy showed difficulty in achieving a proper deformity correction with a tendency to recur. Later, several types of pelvic osteotomy and surgical techniques have been reported and employed by orthopedic surgeons to enhance the approximation of symphyseal diastasis, including bilateral posterior pelvic osteotomies, horizontal pelvic osteotomies, and combined vertical and horizontal pelvic osteotomies [13–15].

However, only a few previous studies focused on long-term outcomes, with only one reported on a specific osteotomy technique [16–18]. Therefore, there’s limited evidence to confirm which osteotomy techniques provide the most suitable and effective outcomes for correcting pelvic deformities in bladder exstrophy over a long-term period. Moreover, almost all of the previously published osteotomy techniques needed fixation, either with k-wires, screws, or an external fixator, which can increase operative time, implant removal operating cost, and risk of infection. Accordingly, in this study, we proposed a new technique of bilateral iliac wing bayonet osteotomies. This osteotomy technique was designed to close pubic symphyseal diastasis without fixation, with an improvement in both external rotation and shortening of the pelvic bone. Our study aimed to describe the surgical technique of bilateral iliac bayonet osteotomies and orthopedic management of bladder exstrophy and to report on the long-term clinical and radiographic outcomes.

## Patients and methods

### Study design and patients

Following the hospital’s institutional review board approval, a retrospective review of patients diagnosed with bladder exstrophy, who underwent bilateral iliac bayonet osteotomies together with the closure of bladder exstrophy between the years 1993 and 2022 was conducted. A total of 28 children, aged 48 months old at the time of surgery were included. Of these, 11 were able to attend a special follow-up clinic or were interviewed by telephone by one of the authors with completed charts and recorded data. The remainder were unable to follow up. Data on the patients’ ages, genders, details of the surgery, and radiographic and clinical outcomes were collected.

### Clinical endpoints

Clinical outcomes included hip pain, limitation of activity, leg-length discrepancy, gait abnormality, foot progression angle, range of motion of the hip, and palpable for symphyseal separation were evaluated. Anteroposterior radiographs of the pelvis taken preoperatively, postoperatively, and at the latest follow-up were analyzed. Pubic symphyseal diastasis was measured as the distance between the two most medial points of the pubic rami. Evidence of hip dysplasia and the union of osteotomy sites were also assessed. Postoperative assessment of diastasis based on the measurements at the latest follow-up.

### Surgical techniques

The bayonet osteotomy technique proposed in this article was designed to improve the external rotation components and shortening by placing the osteotomy at the top of the greater sciatic notch; therefore when internal rotational folding of the distal portion was applied, the inter-radiate distance and the shortening of the anterior segment were corrected to a certain extent to the point of which the lengthening of the anterior segment was not required to reapproximate the pubic symphysis. The step transverse cut at the middle of the oblique osteotomy was to prevent the vertical shifting of the osteotomy. This technique has been developed at our hospital by the senior author (AC) since the year 1993. We improved the technique as the series progressed and now reported the results.

### Osteotomy and redirection

**Fig. 1:**
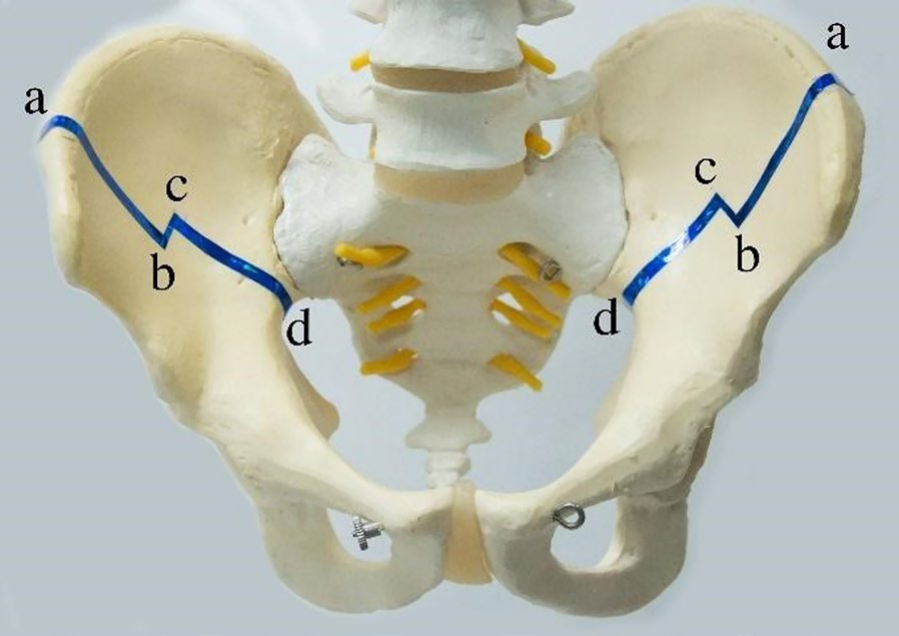
The sawbones model demonstrated the bayonet osteotomy. the first vertical osteotomy started from the iliac crest to the middle of the crest (from **a** to **b**), then the second vertical cut of the distal portion continued down to the greater sciatic notch (from **c** to **d**). The third transverse cut was then osteotomized by using a small curve osteotome (from **b** to **c**)

**Fig. 2:**
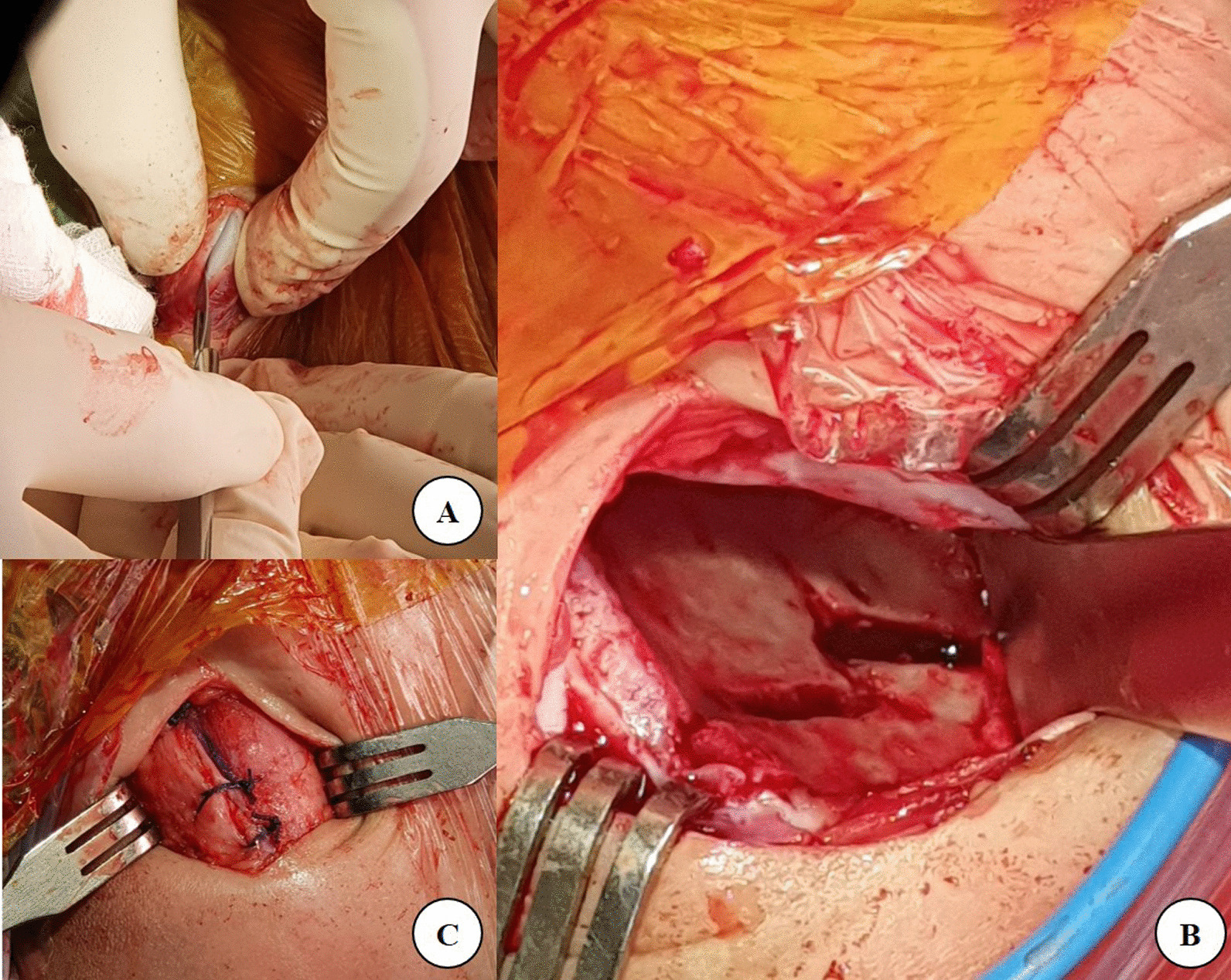
Dissection directed to the apophysis of the iliac crest and sharply split the apophysis in the middle (**A**). The bayonet osteotomy was shown in **B**. After the osteotomy was done, the apophysis was resutured (**C**)

The patient was in a supine position on a radiolucent operating room table under general anesthesia. A fluoroscopy machine with a wide field of view may be used for accurate and safe osteotomy cuts during the case. The incision was carried along the superior portion of the iliac crest approximately one-third of the crest. Dissection was directed to the apophysis of the iliac crest and sharply split the apophysis in the middle by using a number 10 blade. The periosteal was then lifted along with the apophysis from the inner table of the iliac crest and carried along to the greater sciatic notch. In Fig. 1, the vertical osteotomy was carried out starting from the iliac crest to the middle of the crest (from a to b) by using a 3-mm match head high-speed burr and the outer cortex was finished with a small cure osteotome, then cut the distal portion continued down to the greater sciatic notch (from c to d). The transverse portion was osteotomized by using a small curve osteotome (from b to c). Care had to be taken for bleeding of the penetrating branch at the distal portion of the iliac crest inner table, which can be stopped by an electric cautery coagulator and bone wax. Both ends of the osteotomy (the iliac crest and the top edge of the greater sciatic notch) had to be completely cut otherwise the osteotomy site could not be folded (Fig. 2). After the bone cut was completed, the anterior segment of the osteotomy was then folded inward with a gentle push from the hip on both sides to ensure that the osteotomy was complete and foldable. Due to the design of the bayonet cut, the folded iliac wings were stable without any fixations needed.

### Pubic symphysis approximation

**Fig. 3:**
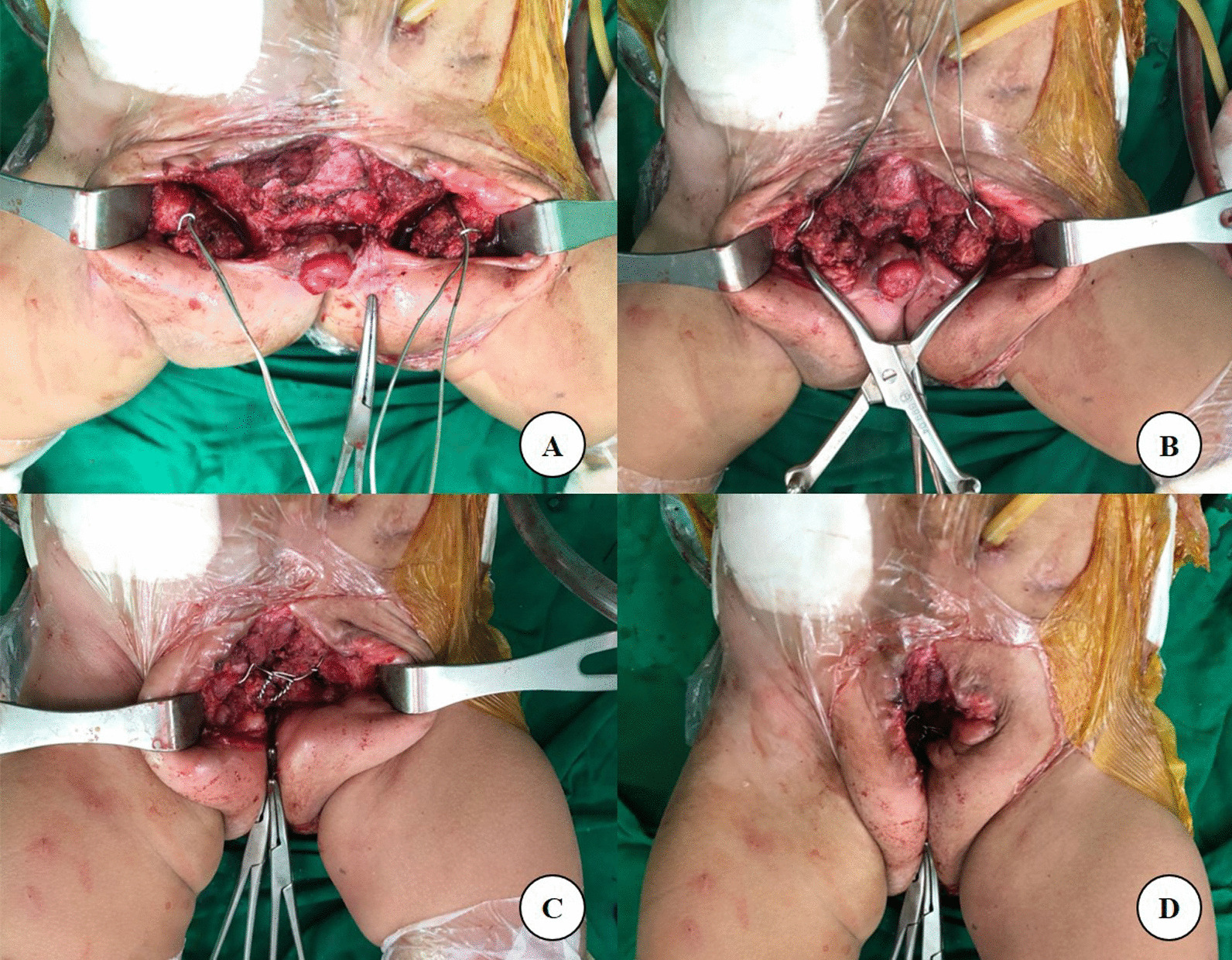
**A**–**D** The pubic symphysis on both sides was identified and dissected. By using a right-angled clamp, a silver wire of number 20 passed around the superior pubic symphysis (**A**). The symphyseal diastasis was approximated temporarily by a Spanish clamp (**B**). Both symphyses were then reapproximated and secured (**C**, **D**)

The second part of the procedure was to reapproximate the pubic symphysis. This was usually carried out after the urologist finished the dissection of the bladder and genital structure at the midline. The pubic symphysis on both sides was identified and dissected. Do not peel off the soft tissue cartilage and periosteal of the pubic symphysis. The symphyseal diastasis was approximated temporarily by a Spanish clamp. By using a right-angle clamp, silver wire of number 20, or other types of suture material, such as Ethibond No. 2, passed around the superior pubic symphysis as shown in Fig. 3. Both symphyses were then reapproximated and secured. Be careful not to injure the urological and genitalia structures and kept the wire underneath the pubic symphysis.

### Postoperative care

**Fig. 4:**
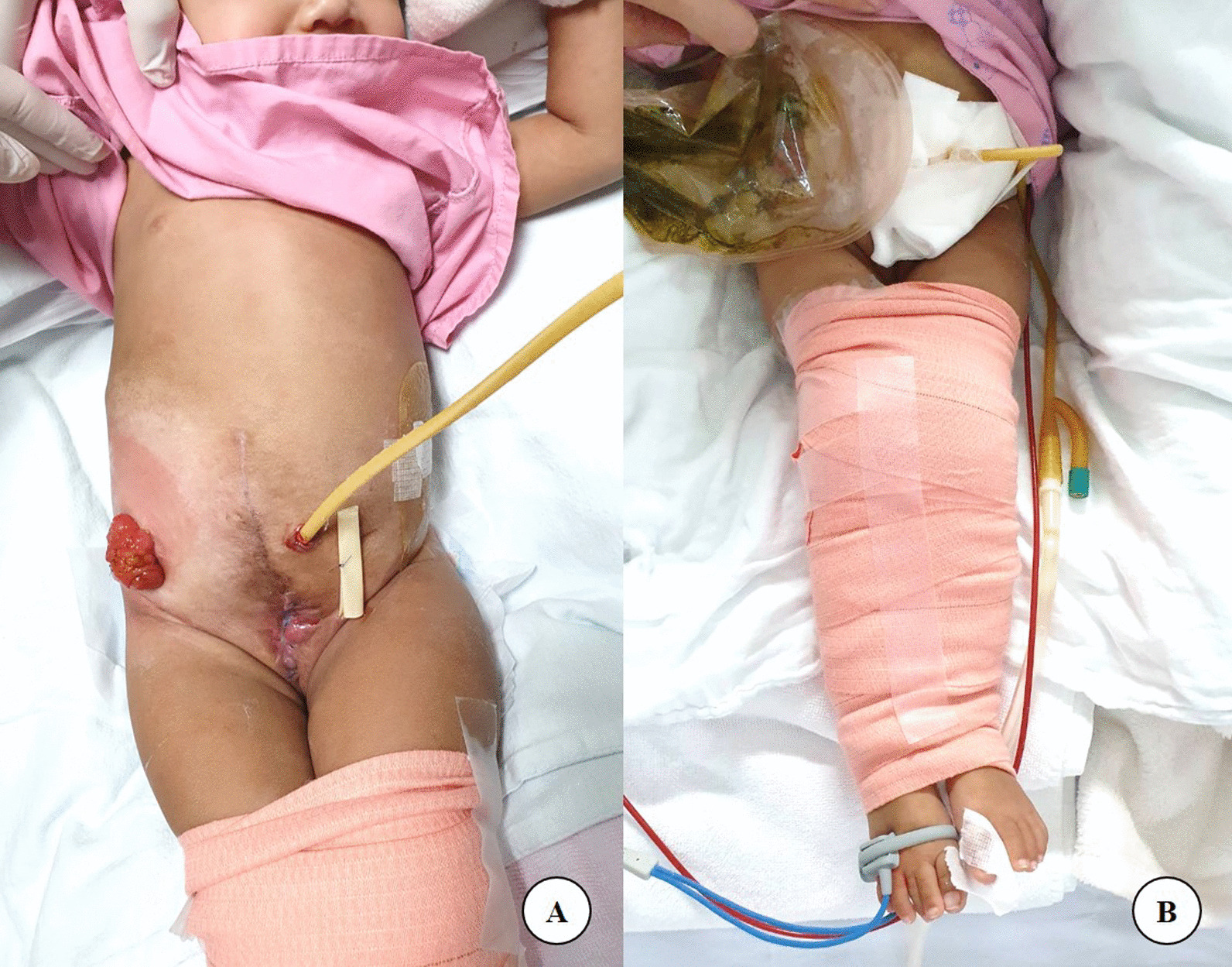
**A**, **B** During the postoperative period, both legs were wrapped in an elastic bandage and soft padding between bony prominence in an adducted and internally rotated position (mermaid wrap) for two weeks (**A**, **B**)

**Fig. 5:**
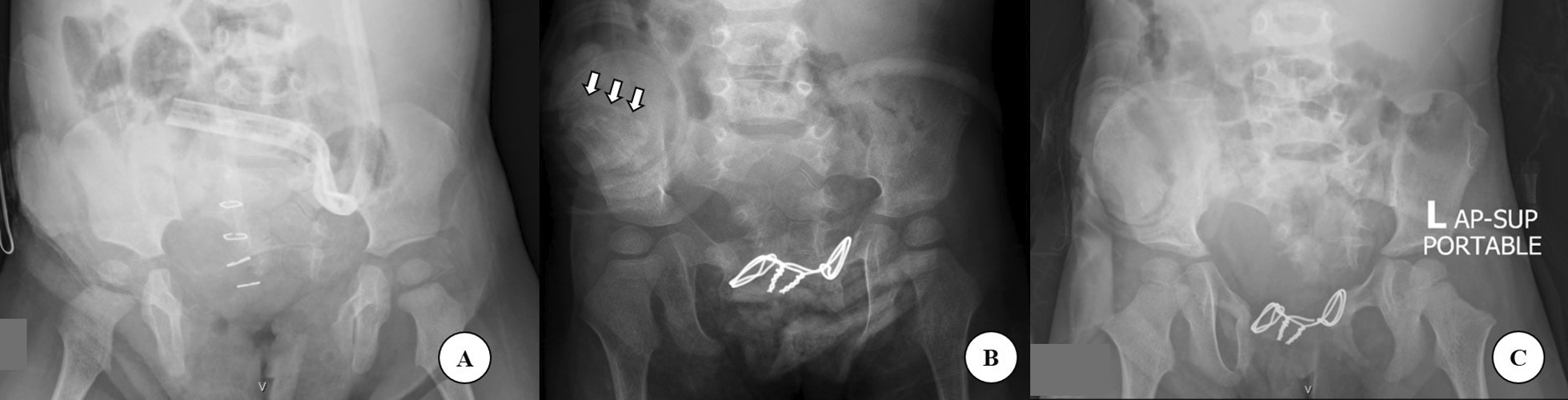
**A**–**C** Radiographs of a 16-month-old girl at preoperative (**A**), postoperative (**B**), and at 3 years follow-up, white arrows emphasized osteotomy sites (**C**), after underwent bilateral bayonet osteotomy and pubic symphysis approximation with wiring

During the postoperative period, both legs were wrapped in an elastic bandage and soft padding between bony prominences, such as the femoral condyles and malleolus, in an adducted and internally rotated position (mermaid wrap) for two weeks until the bony union has achieved. This enabled free handling of wounds and catheters. (Fig. 4) Preoperative and postoperative radiographs after bayonet osteotomy were shown in Fig. 5. The silver wire used for pubic symphysis approximation was removed at one year postoperative.

All procedures performed in the study involving human participants were in accordance with the ethical standards of the institutional and/or national research committee and with the 1964 Helsinki declaration and its later amendments or comparable ethical standards.

### Statistical analysis

Descriptive statistics (mean and standard deviations) were used to describe the participants’ demographic data and postoperative clinical and radiographic outcomes. Statistical calculation was performed using Stata Statistical Software (College Station, TX: StataCorp LLC).

## Results

A total of 11 patients (9 female and 2 male) with an average age at operation was 9.14 ± 11.57 months. The average followed-up time was 14.67 ± 9.24 years. Data on patients’ demographics are summarized in Table 1.

**Table 1:** Demographic data

	Patients (*n* = 11)
Age at time of operation (months)	9.14 ± 11.57 (6–48)
Sex (*n*)	
Male	2
Female	9
Preoperative pubic symphysis diastasis distance (cm)	4.58 ± 1.37 (3.56–6.8)
Postoperative pubic symphysis diastasis distance (cm)	2.05 ± 1.13 (0.83–3.4)
Operative time (mins) (included urologic operation)	354 ± 81.73 (260–420)
Blood loss (ml)	190 ± 62.85 (100–250)
Follow-up time (years)	14.67 ± 9.24 (0.75–29)
Modified Harris Hip score (at the latest follow-up)	90.45 ± 1.21 (88–91)

Data in columns are presented as mean ± SD (range) unless otherwise specified

The average modified Harris Hip score was 90.45 ± 1.21. No patients reported hip pain, limping, or leg length discrepancy. Of 11 patients, four were able to visit the follow-up clinic and perform a physical examination. Radiographically, all patients demonstrated decreased pubis symphyseal diastasis distance after the operation, even though some of them became wider at the latest follow-up visit, but no evidence of any clinical symptoms was associated. All osteotomy sites were completely union and no hip dysplasia was seen. Details on radiographic and clinical outcomes at the latest follow-up visit are shown in Table 2.

**Table 2:** Radiographic and clinical outcomes at the latest follow-up visit

	1	2	3	4
Age (years)	5	9	3	27
Sex	Female	Female	Female	Female
Follow-up time (years)	5	9	2	23
Modified Harris Hip score	88	91	91	91
Pubic symphysis diastasis distance (cm)				
Preoperative	3.56	3.91	5.0	6.8
Postoperative	1.24	1.5	1.4	0.83
At latest follow-up	3.1	5.3	1.6	5.1
*Clinical outcomes*				
Left				
Foot progression angle (°)	ER 10	ER 5	ER 5	ER 10
Hip ER/IR (°)	40/40	70/60	60/50	55/40
Hip flexion/extension (°)	130/10	140/15	100/20	115/10
Hip abduction/adduction (°)	50/20	40/20	35/20	40/25
Right				
Foot progression angle (°)	ER 10	ER 10	ER 5	ER 10
Hip ER/IR (°)	40/40	60/50	60/60	50/40
Hip flexion/extension (°)	120/10	130/20	100/30	120/20
Hip abduction/adduction (°)	45/20	40/20	40/30	40/25

ER = External rotation. IR = internal rotation

No intraoperative complications were found. Postoperative complications were 1 case of superficial wound infection and 2 cases of urinary tract infection. Two cases of bladder erosion (one from wiring and one from Ethibond) which were treated with wire and Ethibond removal at one month postoperative. All patients received proper standard treatment and fully recovered.

## Discussion

The goal of bladder exstrophy surgery was to close the pubic symphyseal gap with a tension-free approximation of the pubic bones and soft tissues in the midline [13, 19, 20]. Management of the gap closure was a patient's age-dependent decision. In the first few hours of life, direct closure without osteotomy could be successfully performed. However, osteotomy was required as pelvic bone malleability markedly decreased afterward [18]. Previous studies concluded that closure of the bladder without pelvic osteotomy showed a higher incidence of premature hip arthrosis, but when combined bladder closure and pelvic osteotomy, it increased the odds of a successful outcome [10, 21, 22].

Several types of pelvic osteotomy and surgical techniques have been reported, including bilateral posterior pelvic osteotomies, horizontal pelvic osteotomies, and combined vertical and horizontal pelvic osteotomies [13–15]. These previously described techniques had pros and cons. Originally proposed anterior pelvic osteotomy was a simple procedure with shorter operative time, no need to change the patient’s position and minimal blood loss [23]. However, the correction of externally rotated pelvic bones could not be achieved. Also, not suitable for older patients with less elasticity of the sacroiliac ligaments, and extreme diastasis (> 6 cm) [24]. Posterior vertical pelvic osteotomy, described by Roberts et al., showed simplified bladder closure and repair of the associated divarication of the rectus abdominis. However, it increased operative time for changing position, increased blood loss, insufficient pelvic closure as well as postoperative pain [19]. In 2001, Sponseller et al. recommended anterior innominate osteotomy with an additional posterior osteotomy to facilitate complete correction of an externally rotated posterior portion of the pelvis in patients older than 2 years of age or with cloacal exstrophy [10]. Conversely, Aly demonstrated that by applying lateral pressure over both greater trochanters after anterior pelvic osteotomy, the distal iliac fragments could be rotated medially to correct the rotated pelvis and closure of symphyseal diastasis without adding posterior pelvic osteotomy [2]. Our technique also confirmed this conclusion.

The bilateral bayonet pelvic osteotomy proposed in this study was designed to close the symphyseal gap and improve both the pelvic external rotation and the pelvic shortening without needing additional posterior pelvic osteotomy, which was a time-consuming procedure and more blood loss. Moreover, other previous osteotomy techniques needed fixation, such as a k-wire or external fixator, to stabilize osteotomy sites [2, 25]. However, in our technique with the bayonet cut, the osteotomy sites locked after folding both iliac wings and produced enough stable bone contact without any k-wire fixation needed. Therefore, there is no need for another k-wire removal surgery, which can increase the risk during additional operations and costs. Our results also showed no surgical technique-related complications such as nerve injury or non-union of osteotomy sites.

**Fig. 6:**
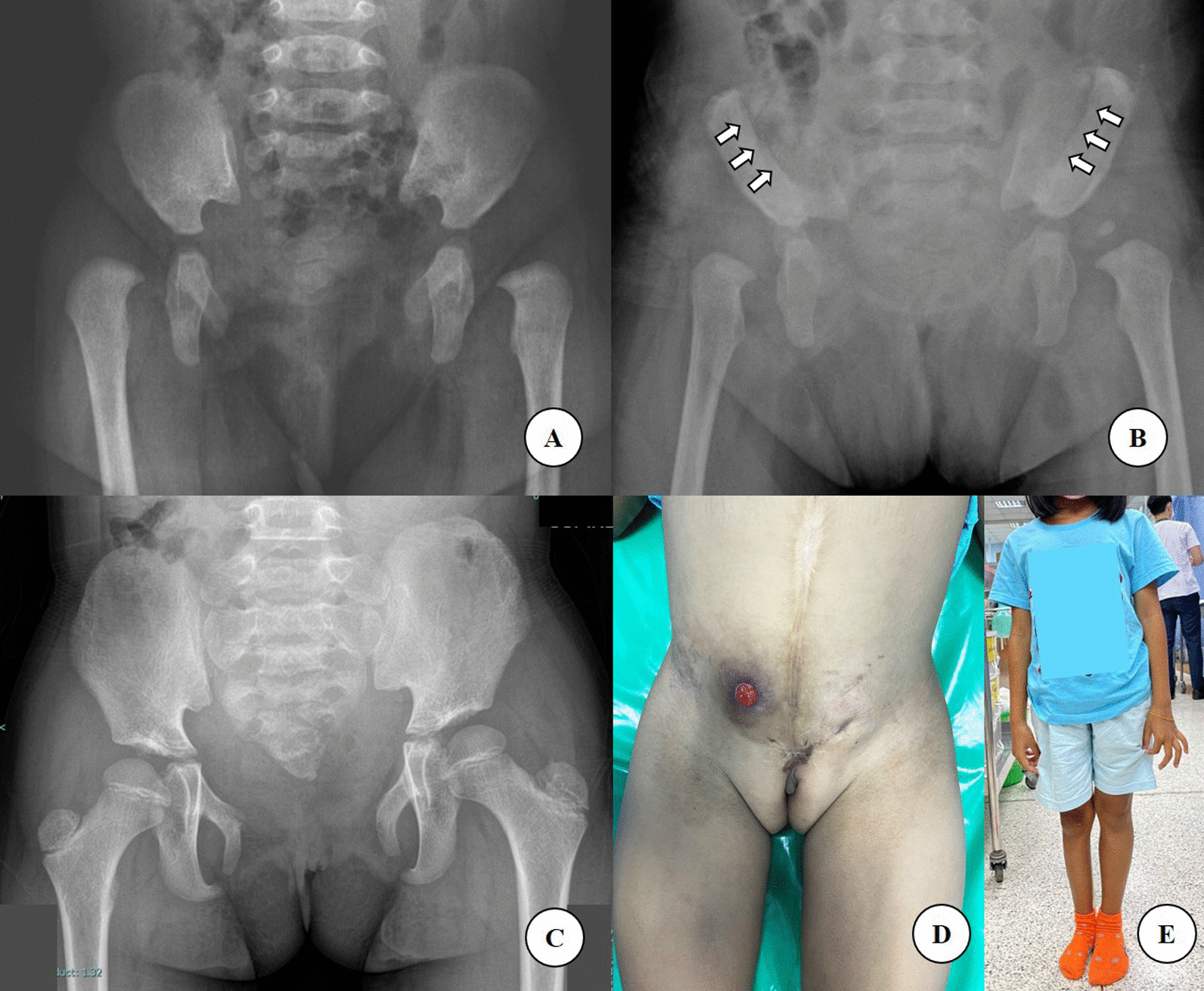
**A**–**E** At 6-month-old radiographs of preoperative (**A**) and postoperative (white arrows emphasized osteotomy sites) (**B**), and at 9 years follow-up (**C**) after underwent bilateral bayonet osteotomy and pubic symphysis approximation with Ethibond. Patient presented with successful bladder closure without any clinical symptoms at 9 years follow-up (**D**, **E**)

Long-term results from our studies demonstrated successful symphyseal diastasis closure without any clinical symptoms. Even though the diastasis gap was wider at the latest follow-up compared to immediate postoperative radiographic findings, no clinical symptoms were affected. These diastasis gaps were healed with fibrous tissue; some can be palpable but showed no association with any clinical outcome (Fig. 6). When rotating both distal iliac bony fragments medially, it will correct the pelvic bone’s external rotation, preventing a wide-based, waddling, and externally rotated gait. Clinically, all patients in our study were within the normal range of foot progression angle, full hip ROM, no hip pain, leg-length discrepancy, or abnormal gait found. Furthermore, all patients had reported an excellent modified Harris Hip score without any limitation to daily living activities.

Our osteotomy technique would benefit older children with less malleability of the pelvic bone and wide diastasis. Long-term results showed improvement both clinically and radiographically. Accordingly, the bayonet osteotomy would be another good option for correcting pelvic deformity.

This study had a limited number of patients due to the rare condition of the disease, and only a small number of patients with long-term follow-up were included. Furthermore, data from some patients were not completely recorded in the past, leading to fewer cases for evaluation.

## Conclusion

The bilateral iliac wings bayonet osteotomies technique demonstrated a safe and successful pubic symphyseal diastasis closure with an improvement both clinically and radiographically. Moreover, it showed good long-term results and excellent patient’s reported outcome scores. Therefore, it would be another effective option for pelvic osteotomy in treating bladder exstrophy patients.

## Data Availability

The datasets used and/or analyzed during the current study are available from the corresponding author on reasonable request.
